# Impact of School Cycles and Environmental Forcing on the Timing of Pandemic Influenza Activity in Mexican States, May-December 2009

**DOI:** 10.1371/journal.pcbi.1004337

**Published:** 2015-08-20

**Authors:** James Tamerius, Cécile Viboud, Jeffrey Shaman, Gerardo Chowell

**Affiliations:** 1 Department of Geographical and Sustainability Sciences, University of Iowa, Iowa City, Iowa, United States of America; 2 Fogarty International Center, National Institutes of Health, Bethesda, Maryland, United States of America; 3 Environmental Health Sciences, Columbia University, New York, New York, United States of America; 4 School of Public Health, Georgia State University, Atlanta, Georgia, United States of America; Duke University, UNITED STATES

## Abstract

While a relationship between environmental forcing and influenza transmission has been established in inter-pandemic seasons, the drivers of pandemic influenza remain debated. In particular, school effects may predominate in pandemic seasons marked by an atypical concentration of cases among children. For the 2009 A/H1N1 pandemic, Mexico is a particularly interesting case study due to its broad geographic extent encompassing temperate and tropical regions, well-documented regional variation in the occurrence of pandemic outbreaks, and coincidence of several school breaks during the pandemic period. Here we fit a series of transmission models to daily laboratory-confirmed influenza data in 32 Mexican states using MCMC approaches, considering a meta-population framework or the absence of spatial coupling between states. We use these models to explore the effect of environmental, school–related and travel factors on the generation of spatially-heterogeneous pandemic waves. We find that the spatial structure of the pandemic is best understood by the interplay between regional differences in specific humidity (explaining the occurrence of pandemic activity towards the end of the school term in late May-June 2009 in more humid southeastern states), school vacations (preventing influenza transmission during July-August in all states), and regional differences in residual susceptibility (resulting in large outbreaks in early fall 2009 in central and northern Mexico that had yet to experience fully-developed outbreaks). Our results are in line with the concept that very high levels of specific humidity, as present during summer in southeastern Mexico, favor influenza transmission, and that school cycles are a strong determinant of pandemic wave timing.

## Author Summary

An influenza pandemic virus emerged in North America in 2009. Although the virus spread worldwide within months of its emergence, the timing of peak pandemic activity varied by nearly a year across global populations. In fact, the most intense period of pandemic activity occurred earlier in temperate countries of the southern hemisphere (e.g., South Africa, Argentina and Australia) than in countries that reported the first infections (Mexico and the US). This indicates that the timing of peak pandemic activity did not directly align with the arrival of the virus in a location, suggesting the influence of social and environmental factors on pandemic influenza transmission. In this study we examined two large pandemic outbreaks (“waves”) that occurred in summer and fall of 2009 in Mexico. The summer wave occurred in the tropical southeastern states of Mexico, whereas the larger fall wave occurred in the central and northern states. We find that the distinct pandemic waves were likely caused by complex interactions between regional differences in specific humidity, population level susceptibility, and school term cycles. Improving our understanding of the factors modulating regional transmission patterns will improve prediction of the spatial and temporal progression of future pandemics.

## Introduction

There is strong evidence that seasonal influenza activity around the world is modulated by environmental variability [1]. Temperate regions are characterized by annual winter epidemics [2,3] that may result from seasonal decreases in specific humidity and subsequent increases in virus survival and transmission [4–6]. Seasonal influenza activity in the tropics is not as clearly phased [7,8], but tends to peak in seasons with high levels of specific humidity and rainfall [1]. Yet, the extent to which these same factors affect the transmission of pandemic influenza remains largely unknown. The emergence of the novel A/H1N1pdm influenza virus in early 2009 and its subsequent global pandemic spread [9] provides a unique opportunity to examine these links.

The influenza A/H1N1pdm virus spread globally within weeks of the first report of a laboratory-confirmed case on April 19, 2009 in southern California [10]. The global dissemination of the virus was facilitated by global transportation networks from its putative source in North America [11], and by June 11, 2009 laboratory-confirmed infections had been identified in over 70 countries [12]. Although the virus had spread worldwide within months of its emergence, the country-to-country timing of peak pandemic activity varied by >40 weeks [13]. In fact, the most intense period of pandemic activity occurred earlier in temperate countries of the southern hemisphere (South Africa, Argentina and Australia) than in countries that reported the first A/H1N1pdm infections (Mexico and the US) [13]. These observations indicate that the timing of peak pandemic activity did not directly align with the arrival of the virus in a location, suggesting the influence of social and environmental factors on pandemic influenza transmission.

Several studies have examined the relative contribution of environmental drivers and school mixing on influenza transmission with conflicting results. School cycles have been shown to play a significant role in the transmission of seasonal influenza by modulating contact rates between children [14,15]. School closures have been linked to reductions in 2009 pandemic A/H1N1 transmission [16–18], while regional variability in school terms and weak child-mobility have been associated with the staggered occurrence of fall pandemic activity in US cities [17,19]. Specific humidity and prior immunity may have played a role in explaining a third wave of pandemic activity during the winter of 2009–2010 in the southeastern US [20]. Similarly, the spatiotemporal patterning of the 2009 pandemic in Canada was associated with school terms and specific humidity [21,22]. In Chile, latitudinal variation in the timing of peak pandemic activity was associated with specific humidity but not with winter vacations, as pandemic activity was already subsiding when schools closed [23]. Altogether, the transmission impact of environmental forcing, population-scale interventions and school cycles has been broached but yet to be fully understood for pandemic influenza.

Here we fit Susceptible-Exposed-Infected-Recovered (SEIR) models to epidemiological data from Mexico using Markov Chain Monte Carlo (MCMC) approaches and investigate the importance of environmental forcing, school cycles, and interventions in generating the observed spatially-heterogeneous waves of 2009 pandemic influenza activity. Mexico is a compelling case study as it encompasses both temperate and tropical regions, interventions and school vacation periods were largely uniform across the country, and spatially-detailed epidemiological records are available [16].

## Methods

### Epidemiological and environmental data

We obtained daily laboratory-confirmed A/H1N1pdm influenza case data for April—December 2009 from a prospective epidemiological surveillance system that was established in response to the pandemic by the Mexican Institute for Social Security [16,24]. These data were previously used to document the regional patterns of pandemic activity in Mexico across 31 states and the Federal District (hereafter we refer to these as 32 “states”) [16]. Circulation of the pandemic virus was intense during 2009 in Mexico, subsided by January 2010, and was followed by a period of 2-years of sporadic transmission [25]. Hence we capture the full extent of the first year of pandemic activity in this analysis.

**Fig 1:**
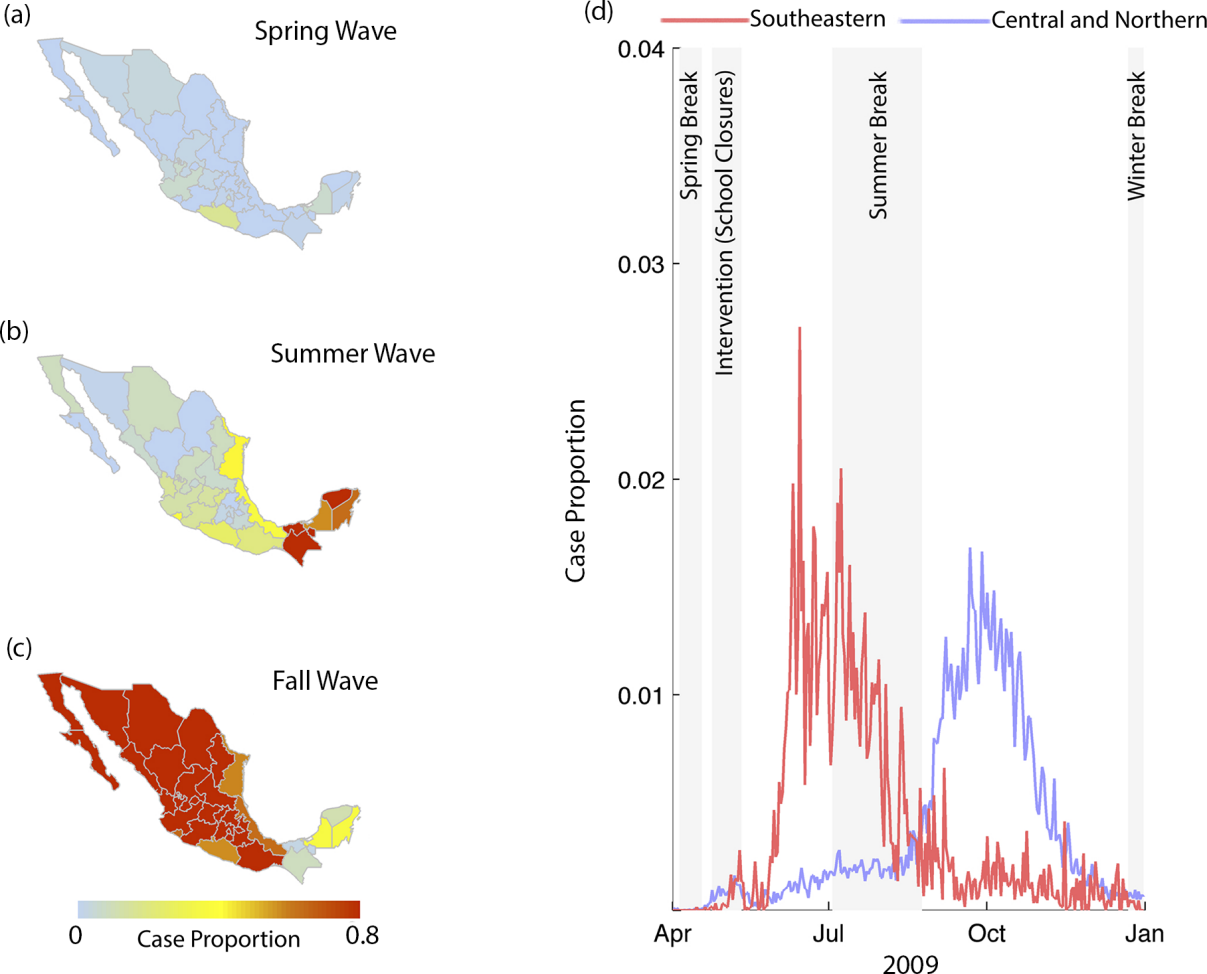
Spatial and temporal structure of pandemic waves. Cumulative case proportions (i.e., the number of infections during a time period divided by the total number of infections during the study period) for the *(a)* spring, *(b)* summer and *(c)* fall waves by state. *(d)* Time series of daily case proportion for each state, and case proportions averaged across southeastern (red), and central and northern states (blue). Altogether, the plots indicate that the spring wave was relatively minor relative to the summer and fall waves; and that the summer and the fall waves were geographically distinct. Cumulative case proportions for the *(a)* spring, *(b)* summer and *(c)* fall waves by state. *(d)* Time series of daily case proportion for each state, and case proportions averaged across southeastern, and central and northern states. The plots indicate that the spring wave was relatively minor relative to the summer and fall waves; and that the summer and the fall waves were geographically distinct.

A mandatory policy of school closure was strictly enforced for a 14-day period during April 27—May 11, 2009 across all states in Mexico [16]. In addition, our study period encompasses three school vacation periods, synchronous across Mexico, including a spring (April 5–18), summer (July 3—Aug 24) and winter break (beginning Dec 22; Fig 1). We retrieved and compiled daily specific humidity, precipitation and temperature data for the study period from the Global Land Data Assimilation System [26] for the most populous city in each state.

### Pandemic patterns and descriptive statistical analysis

There were three spatially-heterogeneous pandemic waves in Mexico in 2009, including a spring wave from April 1—May 20, a summer wave from May 21—August 1, and a fall wave from August 2—December 31, as previously defined [16]. For each state, we calculated the cumulative case proportion of each wave (the number of cases during the wave relative to the total number of cases during the study period) and their association with average temperature, average humidity and cumulative precipitation conditions during each wave period using Pearson correlation. We then classified each location as dominated by a spring, summer or fall wave based on the week with the maximum number of cases.

### Influenza transmission models

To further assess the dynamical effects of environmental variability and school cycles on pandemic influenza transmission, we developed a deterministic SEIR compartmental model at the state level. As a first step, we fit separate models for each state, and as a second step, we explore a meta-population framework allowing for coupling between states. The simplest formulation of our model (independence between states) is expressed by the following equations:

$$\begin{array}{l} dS_{i}/dt=-\lambda_{i}S_{i}\\ dE_{i}/dt=-\lambda_{i}S_{i}-\theta^{-1}E_{i}\\ dI_{i}/dt=\theta^{-1}E_{i}-\alpha^{-1}I_{i}\\ dR_{i}/dt=\alpha^{-1}I_{i} \end{array}$$

where *S*_*i*,_ *E*_*i*,_ *I*_*i*,_ *R*_*i*_ are, respectively, the number of susceptible, exposed, infected, and recovered individuals in Mexican state *i*, *θ* is the mean latency period, *α* is the mean infectious period, and λ_*i*_ is the force of infection [27]. In the main analysis presented here, the mean latency period (*θ*) was fixed at 1.4 days based on past estimates [28], while the mean recovery rate (*α)* was fixed at 1.6 days, consistent with a mean serial interval of 3 days [29]. Further sensitivity analysis was performed in which the mean latency period (*θ*) was allowed to vary from 1–1.8 days, while the mean recovery rate (*α)* was allowed to vary from 0.6–2.6 days, consistent with a mean serial interval of 2.4–3.6 days [29].

We allowed transmission to vary spatially and temporally as a function of specific humidity, school terms and interventions. Following [6] and [20], we let the basic reproduction number, *R*_*0*,*i*_ *(t)*, vary with time as a function of daily state-specific specific humidity *q*_*i*_ *(t)*:

$$R_{0,i}(t)=f(q_{i}(t))$$

**Fig 2:**
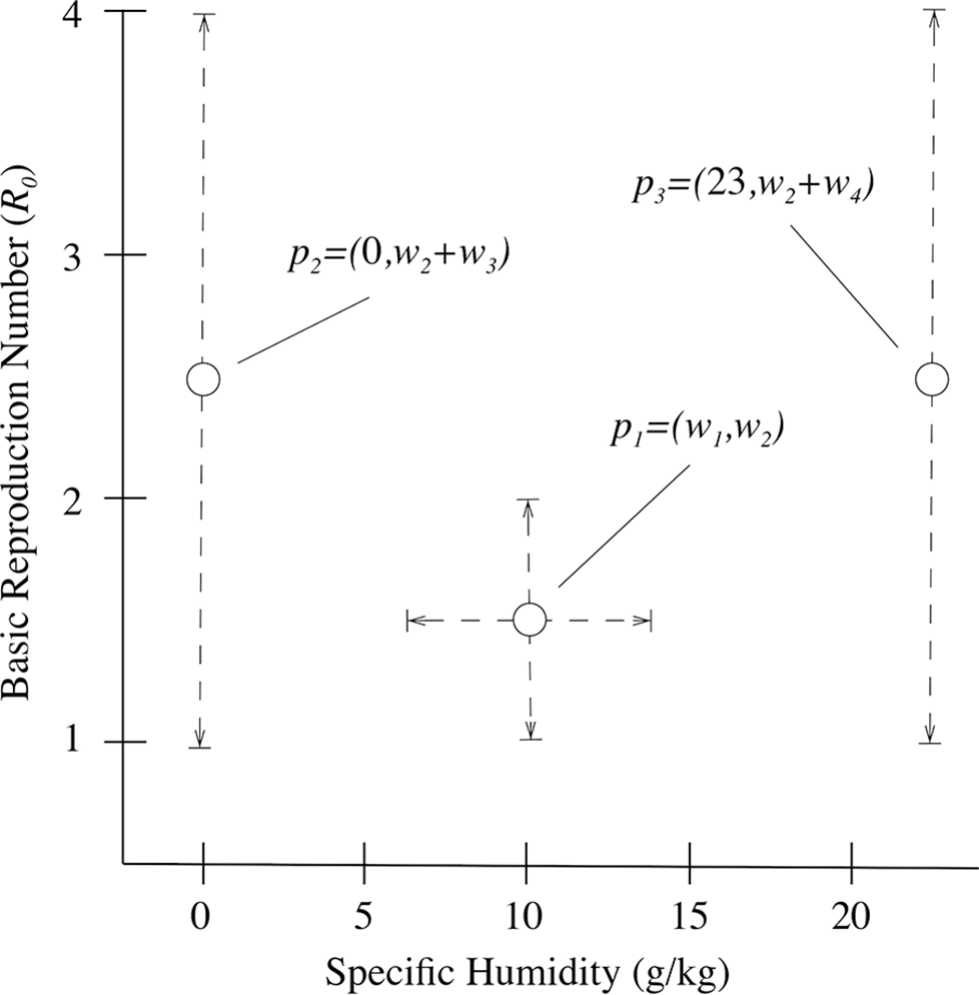
Specification of *R*_*0*_. A diagram showing the three critical points (*p*_*1*_, *p*_*2*_, *p*_*3*_) used to define the relationship between specific humidity and *R*_*0*_. The points were allowed to vary across indicated ranges (dashed lines). The central point, *p*_*1*_, was allowed to vary along the x- and y-axes; whereas *p*_*2*_ and *p*_*3*_ only varied along the y-axis as 0 and 23 g/kg were the bounds of specific humidity.

We chose this formulation for *R*_*0*_ *(t)* because environmental forcing was the main factor under investigation in this study. The functional relationship between *R*_*0*_ and specific humidity was defined by fitting a Piecewise Interpolating Polynomial (PCHIP) curve through three critical points (*p*_*1*_, *p*_*2*_, *p*_*3*_). PCHIP was used because it provided optimal control over the relationship by forcing the curve to pass through the specificed points without overshooting the defined minima or maxima. The critical points were defined by 4 free parameters (*w*_*1*_, *w*_*2*_, *w*_*3*_, *w*_*4*_ *)* (Table 1). We specified that *R*_*0*_ values at *p*_*2*_ and *p*_*3*_ to be greater than or equal to *p*_*1*_ (Fig 2). Altogether, this specification of the points allowed for U-, J-, and L-shaped cruves, in addition to flat lines (see S1 Text).

**Table 1:** A description of the epidemiological parameters for each model. The references indicate the studies that informed the paramter estimates and ranges. The “x” indicates parameters that are included in the corresponding model.

Parameter	Description	Refs.	Range	Model 1 (No spatial coupling, independent fit to each state)	Model 2 (meta-population model with coupling with edjacent states)	Model 3 (meta-population model with coupling with edjacent states and greater Mexico City hub)
*w*_*1*_	Value of *specific humidity* (g/kg) corresponding to minimum *R*_*0*_	[1]	6–14	x	x	x
*w*_*2*_	Minimum *R*_*0*_	[6]	1–2	x	x	x
*w*_*3*_	Added to *w*_*2*_ to define *R*_*0*_ for *specific humidity* = 0 g/kg	[6]	0–2	x	x	x
*w*_*4*_	Added to *w*_*2*_ to define *R*_*0*_ for *specific humidity* = 23 g/kg	[1]	0–2	x	x	x
γ_*1*_	Transmission efficiency during school vacation	[15]	0.55–0.90	x	x	x
γ_*2*_	Transmission efficiency during intervention and school closures	[15, 16]	0.55–0.90	x	x	x
*τ(0)*	Rate of infected population at time = 0	NA	10^−6^–10^−3^	x	x	x
*c*_*adj*_	Links force of infection between adjacent states	NA	0–1	—	x	x
*c*_*hub*_	Links force of infection in hub states with all other states	NA	0–1	—	—	x
*μ(0)*	Pre-pandemic population susceptibility fraction	[32]	0.95	x	x	x
*Θ*	Latency period (days)	[28]	1.4	x	x	x
α	Mean infectious period (days)	[28, 29]	1.6	x	x	x

Next, we let the effective reproduction number, *R*_*e*_ *(t)*, depend both on *R*_*0*_ *(t)* the proportion of the susceptible population, *S*_*i*_ *(t)*, as well as school terms, *v(t)* and interventions, *z(t*). For school terms and interventions, we used step functions to represent changes in influenza transmissibility, as follows:

$$\begin{array}{c} v(t)=\{\begin{array}{cccc} 1, & if & \text{school term} & \\ \gamma_{1}, & if & \text{school vacation} & 0.55\leq \gamma_{1}\leq 0.90 \end{array}\\ z(t)=\{\begin{array}{cccc} 1, & if & \text{no intervention} & \\ \gamma_{2}, & if & \text{intervention} & 0.55\leq \gamma_{2}\leq 0.90 \end{array} \end{array}$$

where γ_*1*_, and γ_*2*_ are independent and bounded parameters (Table 2). Altogether, the effective reproduction number (*R*_*e*_) in state *i*, follows:

$$R_{e,i}(t)=v(t)z(t)R_{0,i}(t)S_{i}(t)N^{-1}$$

**Table 2:** Summary of goodness-of-fit measures for select models. The table indicates the number of summer waves accurately predicted in the 6 states in which they were observed, the number of fall waves accurately predicted in the 32 states observed, and the AIC for each model. The parameters included in each model are described in Table 1.

Model	Summer Wave Predicted/Observed	Fall Wave Predicted/Observed	AIC
Model 1 (No spatial coupling, independent fit to each state)	6/6 (100%)	23/26 (88%)	-93805
Model 2 (Meta-population model with coupling with edjacent states)	2/6 (33%)	26/26 (100%)	-93267
Model 3 (Meta-population model with coupling with adjacent states and greater Mexico city hub)	3/6 (66%)	24/26 (92%)	-94165

We also developed several flavors of meta-population models to explore travel effects. Based on past work indicating the importance of local diffusion on influenza transmission [19] we allow for neighboring states to affect the local force of infection. In the absence of mobility data from Mexico to calibrate a more detailed population model, we also allow for the greater Mexico City area to affect the risk of transmission in other areas, as the Mexican capital is a hub for both air and bus travel, the two dominant modes of transportation in the country. Our approach borrows from the concept of gravity models [30], whereby both large populations and nearby populations may affect the risk of dissemination to a new locale.

Specifically, we allow the force of infection in each state, *λ*_*i*_, to be modified by the force of infection in neighboring states, *λ*_*adj*_, and in two “hub” states (Mexico City and the Federal District), *λ*_*hub*_. For each state, *i*, the *λ*_*i*_ at time *t* prior to mixing between states is defined as:

$$\lambda_{i}(t)=B_{i}(t)I_{i}(t)N^{-1}$$

where *B*_*j*_ is the transmission rate for state *i*. *B*_*i*_ *(t)* and *R*_*0*,*i*_ *(t)* are related by:

$$B_{i}(t)=R_{0,i}(t)\alpha^{-1}$$

When both travel effects are included, the force of infection in state *i* is modified by the force of infection in all adjacent states and two hub states as follows:

$${\hat{\lambda }}_{i}(t)=\lambda_{i}(t)+c_{\text{adj}}\sum_{j=1}^{}\lambda_{\text{adj}}(t)+c_{\text{hub}}\sum_{k=1}\lambda_{\text{hub}}(t)$$

where *c*_adj_ and *c*_hub_ are free parameters that are allowed to vary from 0–1.

Overall, we compare the fit of 3 increasingly complex spatial models: (a) models fitted independently to each state (*c*_*hub*_ = *c*_*adj*_ = 0), (b) meta-population models with nearest neighbors coupling (*c*_*adj*_ *>* = 0 and *c*_*hub*_ = 0) and (c) meta-population models with nearest neighbor coupling and hub centered around the capital (*c*_*hub*_ *>* = 0 and *c*_*adj*_ *>* = 0).

All models incorporate estimates of state population size from the National Council of Population, Mexico for 2009 [31]. Populations in each state were assumed to be fully mixed. The initial proportion of susceptibles, *μ(0)*, was set to 95% across all states based on the age structure of the population in Mexico and estimates of worldwide prevalence of age-specific pre-pandemic cross-reactive antibody responses to A/H1N1pdm [32]. As a sensitivity analysis, the initial proportion of susceptible population was allowed to vary from 0.75–0.95. Further, in a separate model we allowed for spatial variation of *μ(0)* between southeastern states and central and northern states (see S1 Text). In all models, initial incidence in the simulations, *τ(0)*, was uniform across states and allowed to vary from 1–1,000 per 100,000 people (Table 2).

The SEIR models were continuous and deterministic. The ordinary differential equations were solved numerically using Matlab version 8.2 (The Mathworks, Inc). We employed an adaptive Metropolis-Hastings algorithm to perform MCMC simulations and estimate parameter values [33]. We estimated model parameters (Table 2) by fitting the model-derived daily case proportion to empirical data for all states during the pandemic period. Using daily case proportion rather than case incidence allows standardization for potential reporting differences between states. We assumed uniform prior distributions for estimated parameters. We allowed the algorithm to run for 100,000 iterations following an initial burn-in of 250,000. The Geweke diagnostic method was employed to assess convergence of chains [34] with values close to 1 deemed satisfactory.

We compared the fit across models with the Akaike information criterion (AIC) using the observed and simulated daily time series across all states. We also assessed model fit by examining whether peak pandemic incidence in each state occurred during the summer or fall in simulated data, and how this corresponded to the observed timing using the chi-square test.

## Results

### Multiple pandemic waves in Mexico: Descriptive analysis

There were three pandemic waves in Mexico during the 2009 pandemic (Fig 1). The spring wave was relatively minor and concentrated in the greater Mexico City area. The summer wave was predominant in southeastern states, and the fall wave was concentrated in central and northern states.

**Fig 3:**
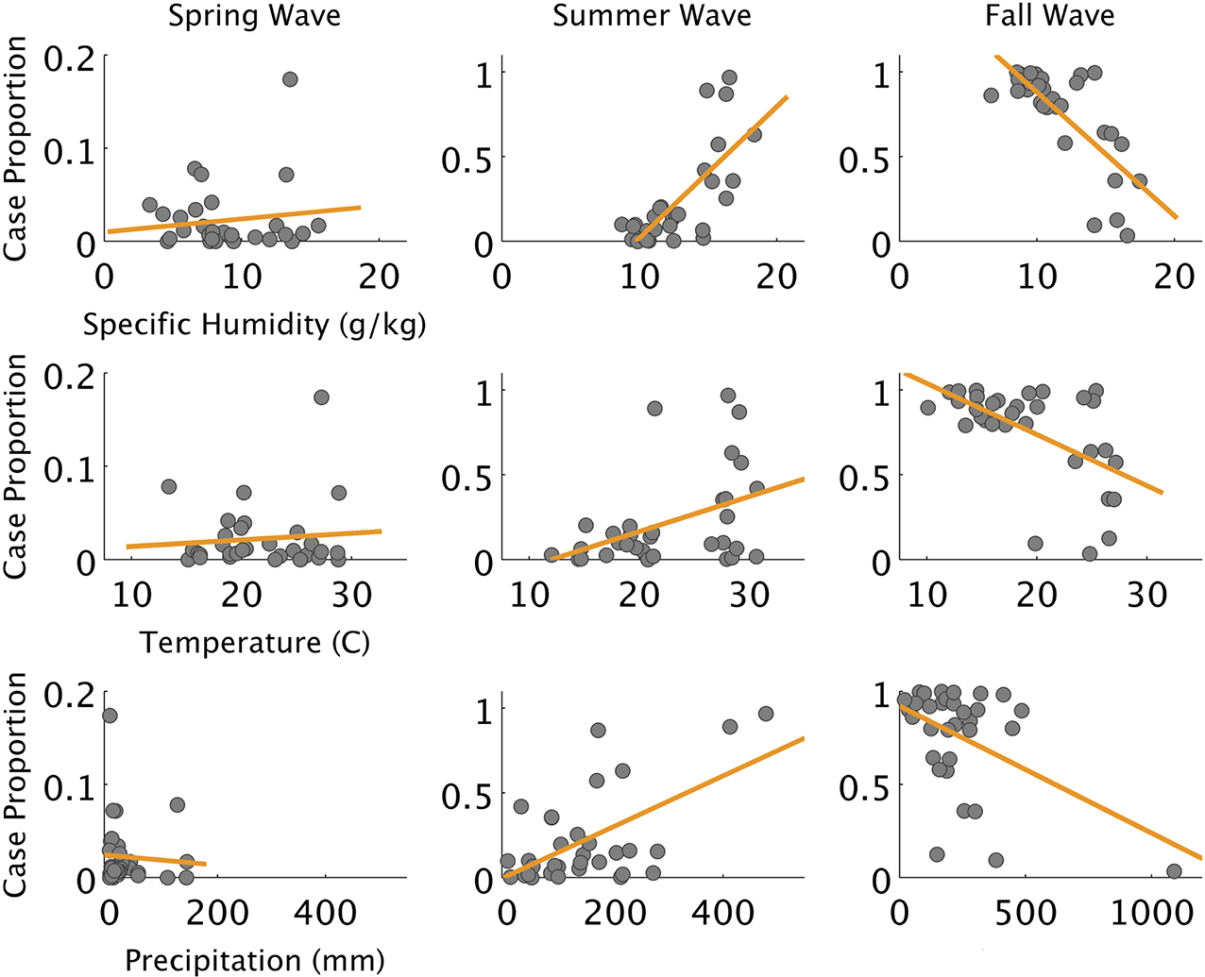
Influenza cases and environmental variables by wave. Relationships between cumulative case proportion for spring, summer and fall waves and environmental variables. Strong relationships are observed between cumulative case proportions and environmental variables during the summer and fall waves; however, the relationship with precipitation is strongly influenced by outliers. Specific humidity has the strongest and most robust relationship for summer and fall waves. The relationships between the environment and the summer wave are opposite of those observed during the fall wave, likely due to spatial heterogeneity of population level susceptibility caused by the summer wave.

The cumulative case proportion during the summer wave was associated with higher mean specific humidity conditions (Pearson’s correlation, r = 0.74, 95% CI: 0.63, 0.85). Mean temperature (r = 0.42, 95% CI: 0.20, 0.63) and rainfall (r = 0.59, 95% CI: 0.20 0.78) were also significantly associated with summer cumulative case proportion, but were strongly influenced by outliers (Fig 3). The cumulative case proportion during the fall wave was negatively associated with specific humidity (r = -0.75, 95% CI: -0.86, -0.62), temperature (r = −0.48, 95% CI: -0.73, -0.37) and precipitation (r = -0.56, 95% CI: -0.70, 0.01). The significant association between precipitation and cumulative case proportion was primarily due to a single outlier (Fig 3).

### Transmission models

We fit mechanistic transmission models to the progression of the pandemic in 32 states to assess the dynamical consequences of school cycles, social distancing interventions (that include but are not limited to school closure, see Fig 1), and spatiotemporal variation in environmental drivers. Since the strongest statistical association between influenza activity and environmental forcing was seen with specific humidity in exploratory analyses (Fig 3), we allowed *R*_*0*_ to vary flexibly as a function of specific humidity only. This aligns with laboratory and epidemiological evidence linking influenza activity with variations of specific humidity [1,5,6,20–23].

**Fig 4:**
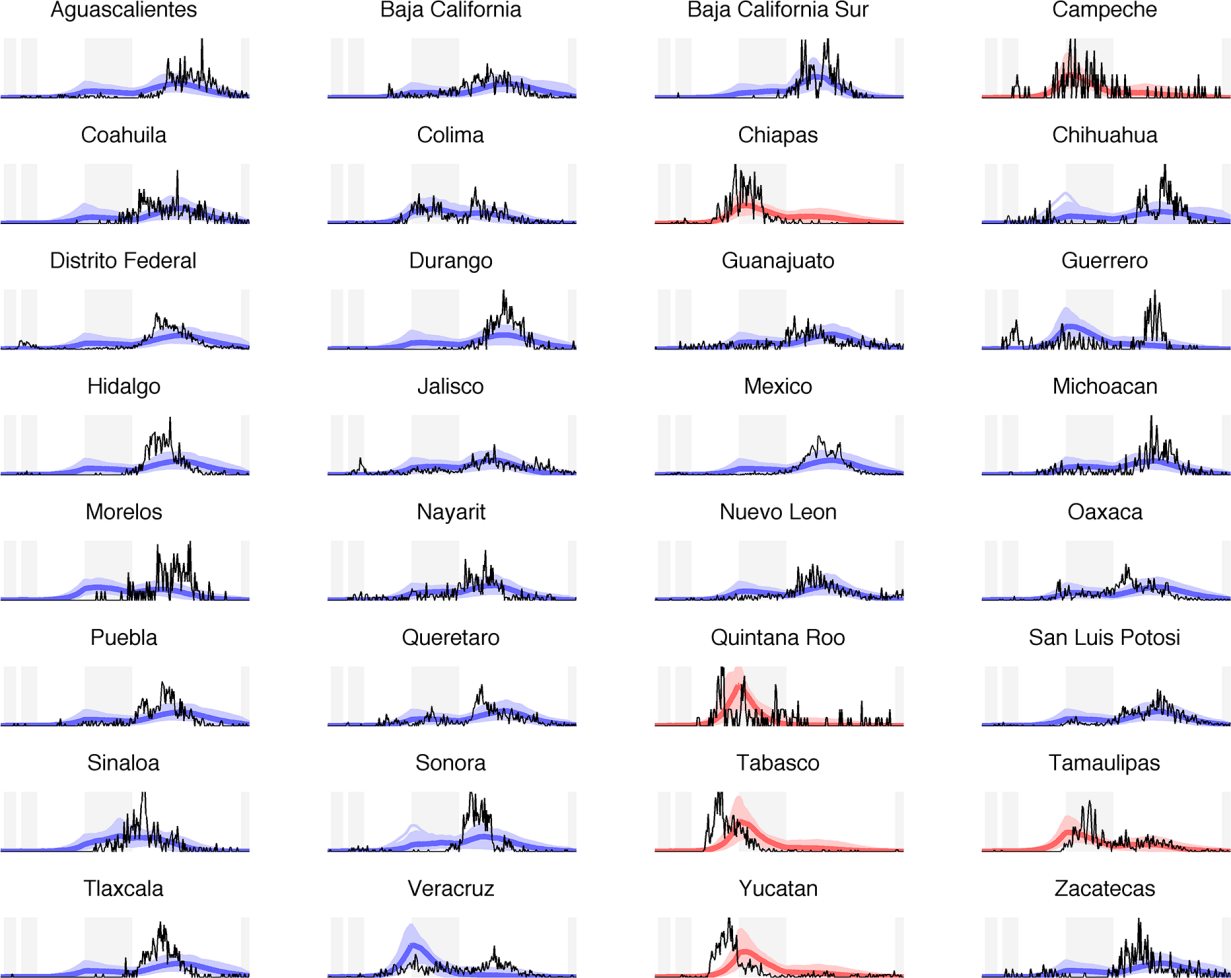
Model results. Time series of case proportion from April 1—December 31 for each state in Mexico during 2009 pandemic. The light blue/red lines correspond to 5000 simulations from random draws from the posterior distributions of each parameter. The dark blue/red lines corresponds to the best-fit simulation based on AIC. The black lines correspond to observed case proportions, and the shaded areas in background correspond to spring vacation, period of school closures and intervention measures, summer vacation, and winter vacation, respectively.

We considered a series of increasingly complex models that included different levels of spatial mixing. Simple transmission models including humidity, school and intervention terms, but no spatial coupling accurately identified the season of the largest pandemic outbreak in 29 of 32 states (P < 0.001; Table 2, model 1). The model also accurately described observed pandemic activity in Colima, where a relatively balanced proportion of cases occurred in summer and fall. However, the model overestimated early summer transmission in several central and northern states, in particular, Veracruz, Guerrero and Morelos where cases primarily occurred during the fall (Fig 4).

Comparison of AIC values across model structures indicated a significant improvement with the addition of a spatial mixing term allowing for interaction between adjacent states; however the proportion of peak incidence correctly predicted with respect to season decreased (27/32; Table 2, model 2). Inclusion of a term representing connectivity with Mexico City did not improve model fit nor peak prediction accuracy (Table 2, model 3). The estimated relationship between *R*_*0*_ and specific humidity, and the effect of school terms and interventions, were similar across all models. Since the model without spatial mixing best predicted pandemic activity, and estimates of the majority of other parameters did not change significantly with inclusion of spatial mixing terms, we focus on the model with no spatial mixing in subsequent sections.

Our MCMC estimation algorithm achieved high convergence with the exception of two parameters, *w*_*1*_ and *τ(0)* which determine the value of specific humidity at which *R*_*0*_ becomes minimum and the initial incidence of infection, respectively (Table 2 and S1 Text). For the best-fit model, the simulated infection attack rate averaged across all states was 28% and ranged from 26–34%.

Models where the mean latency period (*θ*), the mean recovery rate (*α)* and the initial level of susceptibility *μ(0)* were allowed to vary were not as robust as the primary models; however the models suggested a strong and consistent role for the effect of specific humidity and school terms on the pandemic waves (see S1 Text).

### The impact of specific humidity and susceptibility on the occurrence of summer and fall waves

**Fig 5:**
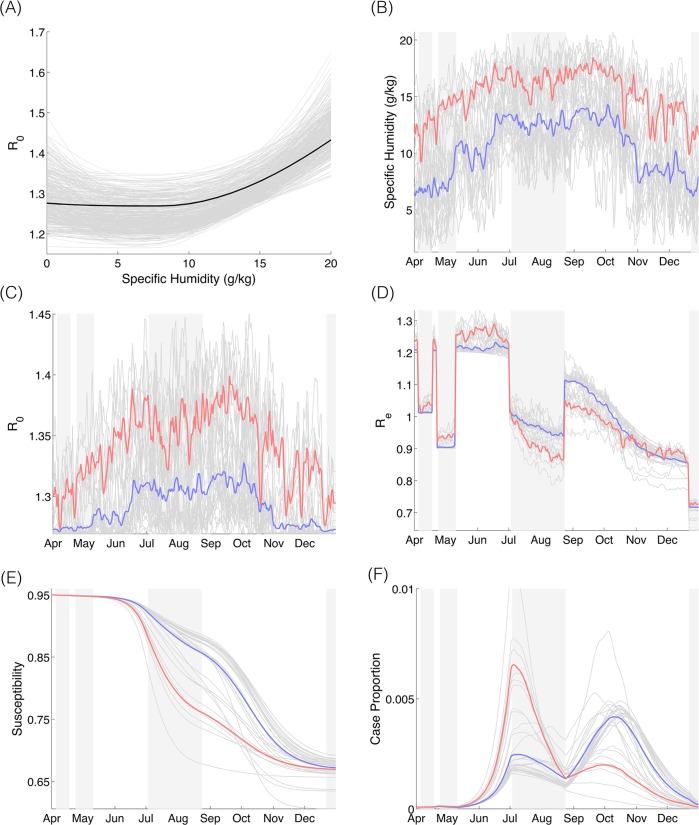
Variability of *R*_*0*_ and *R*_*e*_ during May-December 2009 as a function of changes in specific humidity, interventions, school cycles, and susceptibility, and the resulting impact on pandemic influenza activity, as predicted by the model. (*A*) The relationship between specific humidity and *R*_*0*_. The gray lines are 500 samples generated from the posterior distributions, and the black line corresponds to the mean value of the posterior means. (*B*) The time series of average specific humidity for central and northern states (solid line) and southeastern states (dashed line). (*C*) Time series of *R*_*0*_ for best-fit parameter combination. The step features are related to spring break, the intervention period, summer break and winter break, respectively. (*D*) Time series of simulated *R*_*e*_. (*E*) Time series of simulated population level susceptibility. (*F*) Time series of simulated case proportions. For *B-F*, shaded areas in background correspond to spring vacation, period of school closures and intervention measures, summer vacation, and winter vacation, respectively. The solid lines correspond to the central and northern states, and the dashed lines correspond to the southeastern states. The simulated values were generated using the mean value of the posterior means for each parameter.

The estimated relationship between specific humidity and *R*_*0*_ was J-shaped, with greatest *R*_*0*_ at high levels of specific humidity and minimal *R*_*0*_ at moderate levels of specific humidity (Fig 5; Table 3). Our estimated *R*_*0*_ values ranged between 1.14 and 1.26, depending on specific humidity conditions.

**Table 3:** Parameter estimates and corresponding confidence intervals, and the Geweke index are provided for models with and without mixing.

Parameter	Model 1: no spatial coupling		Model 2: spatial coupling with adjacent states		Model 3: spatial coupling with adjacent states and greater Mexico City hub	
	Parameter Estimate (95% CI)	Geweke	95% CI	Geweke	95% CI	Geweke
*w*_*1*_	7.12 (6.02, 9.60)	0.18	7.81 (6.07, 10.44)	0.08	7.35 (6.01, 9.49)	0.04
*w*_*2*_	1.28 (1.24, 1.40)	0.90	1.59 (1.14, 1.76)	0.79	1.20 (1.01, 1.43)	0.66
*w*_*3*_	0.02 (0.00, 0.08)	0.83	0.10 (0.00, 0.40)	0.76	0.34 (0.10, 0.65)	0.94
*w*_*4*_	0.16 (0.08, 0.26)	0.93	0.43 (0.21, 0.95)	0.82	0.84 (0.50, 1.12)	0.67
γ_*1*_	0.86 (0.80,0.89)	0.98	0.81 (0.76, 0.86)	0.97	0.80 (0.75, 0.85)	0.95
γ_*2*_	0.80 (0.60, 0.89)	0.98	0.79 (0.63, 0.89)	0.88	0.82 (0.65, 0.90)	0.99
*τ(0)*	1.14e-4 (1.05e-4, 1.36e-4)	0.57	1.24e-5 (4.19e-6, 2.06e-5)	0.88	9.49e-6 (3.10e-6, 1.67e-6)	0.30
*c*_*adj*_	—	—	0.01 (0.00, 0.04)	0.68	0.03 (0.01, 0.05)	0.48
*c*_*hub*_	—	—	—	—	0.01 (0.00, 0.02)	0.84
*μ(0)*	0.95	—	0.95	—	0.95	—
*Θ*	1.4	—	1.4	—	1.4	—
α	1.6	—	1.6	—	1.6	—

Our model suggests that population level susceptibility was uniformly high and near initial levels at the beginning of the summer wave (Fig 5). In late May and June 2009, *R*_*e*_ was estimated to be greatest in the southeastern states (mean = 1.26) due to more humid conditions; whereas in the central and northern states *R*_*e*_ was slightly lower (mean = 1.21) due to moderate levels of specific humidity. The model implies that these regional differences in estimated *R*_*e*_, driven by differences in specific humidity, were critical for the formation of the heterogeneous pandemic wave pattern and fomented greater incidence during summer in the more humid southeastern states.

During the summer vacation period, estimates of *R*_*e*_ decreased below 1 in nearly all states owing to school closure. At the beginning of the school term in late August 2009, estimated *R*_*e*_ averaged 1.11 for the majority of central and northern states, where susceptibility remained high (~85%). In contrast, susceptibility estimates in southeastern states were significantly lower (~75%), which reduced transmission rates (Fig 5E).

### Effect of school vacation and social distancing on the occurrence of summer and fall waves

We estimate that school vacation decreased influenza transmission by 14% (95% CI: 10%, 19%) on average during the spring and summer breaks. During the 14-day period in May 2009 when stringent social distancing interventions were put in place, *R*_*0*_ was reduced by 20% (95% CI: 11%, 40%); the large 95% credible interval likely has to do with a strong correlation between estimates of *w*_*2*_ (minimum of *R*_*0*_) and *z* (impact of interventions) in the model. Although we put constraints on the impact of intervention and school vacation periods based on past studies (Table 2 and [15,16]), relaxing these constraints did not change our estimates, attesting to the importance of reduced transmission during these periods. Indeed, simulations indicate that if the summer vacations were eliminated, peak pandemic activity would have occurred in June-July 2009 for all states (see S1 Text).

## Discussion

Our results indicate that the spatiotemporal structure of the 2009 influenza pandemic in Mexico can be understood by the interplay between specific humidity, school cycles, and susceptibility. Our results suggest that high specific humidity in the southeastern states allowed for relatively high transmission rates in late May-June 2009 and favored a substantial outbreak in these states (Fig 5); the pandemic wave in the southeastern states subsided during summer school vacations as *R*_*e*_ decreased. The results suggests that when school activities resumed in the fall, transmission increased due to increased contact rates and triggered outbreaks in some southeastern states, but these were relatively minor due to reduced levels of susceptibility following the summer wave. This is similar to the process that explains differences in the progression of the pandemic across regions in the US during the fall and winter of 2009–2010 [20].

Our model predictions indicate that central and northern states experienced more than 85% of their cases during the fall wave, consistent with observations. Our analysis suggests that the lack of substantial viral activity in this region in late May-June 2009 was due to slightly lower transmission rates associated with moderate levels of humidity, then compounded by school vacation, further reducing transmission rates. When summer vacation ended, the subsequent increase in contact rates made large outbreaks possible in this region. Our simulations suggest that a large summer wave would have occurred in the central and northern states if summer vacation had not reduced transmission (see S1 Text). This highlights that both environmental variability and school cycles were critical for generating the distinct summer and fall wave patterns observed in Mexico.

We developed an SEIR model framework that allowed local levels of specific humidity to modulate transmission rates across a wide distribution of relationships, including a bimodal relationship indicating that influenza activity is enhanced by very low levels and very high levels of specific humidity [1]. Unlike previous studies that examined the effect of specific humidity (among other variables) on the progression of pandemic waves in the US [20], Canada [21,22], and Chile [23], it was necessary to use a bimodal relationship here because Mexico encompasses both temperate and tropical regions.

Previous studies have suggested that population mixing across states may have been a factor in the formation of the spring, summer and fall waves [35]. We developed a model that allowed for mixing between adjacent states and a “hub” region representing the greater capital area (Mexico and the Federal District). However, we found no evidence that connectivity with Mexico City, and hence a hierarchical pattern of spread, could explain the spatiotemporal structure of the pandemic waves. In contrast, connectivity with adjacent states was important, which is reminiscent of the slow diffusive pattern of 2009 pandemic in US cities [19]. This suggests that only local environmental conditions, social mixing and susceptibility patterns shaped the trajectory of the outbreak once the virus became established in the population.

We found no relationship between environmental conditions and the intensity of the spring wave of the 2009 pandemic in Mexico, which remained focused in central states. The spring wave may have only materialized in states highly connected to the geographic origin of the A/H1N1pdm virus prior to the initiation of intervention measures. We are unable to test this hypothesis further as the origins of the 2009 pandemic remain debated.

We estimated a number of disease parameters that compare favorably with independent information, reinforcing the validity of our modeling approach. In particular, our estimates of pandemic infection attack rates ranged from 26–34% for the cumulative period April-December 2009, which is commensurate with estimates derived from global serological surveys (both symptomatic and asymptomatic infections) [29]. Our estimated *R*_*0*_ values, ranging between 1.14 and 1.26, align with estimates from previous studies in Mexico and elsewhere [9,21,28]. However, our estimated *R*_*e*_ values in the southeastern (1.2, summer) and in central-northern states (1.1, fall) are smaller than previous estimates for these regions, which may stem from different modeling assumptions [16]. Finally, we estimated that drastic social distancing interventions reduced transmission by 20% (95% CI: 14%, 40%) in Spring 2009 in Mexico while summer school vacations reduced transmission by 14% (95% CI: 10%, 19%), which is broadly consistent with previous estimates [16].

Our model is prone to a number of limitations. In the absence of prior information on baseline pre-pandemic immunity in Mexico, we considered prior immunity in baseline models to be spatially homogenous at 5%, informed by global age-specific serosurveys [29]. We did not explicitly incorporate the role of heterosubtypic immunity in mitigating the spread of the virus [22,36,37]. Indeed, previous modeling studies suggest that prior immunity from seasonal strains can inhibit the transmission of pandemic strains, thereby either delaying pandemic waves until immunity wanes or creating multi-wave patterns in a single population [38,39]. It is possible that prior immunity in Mexico varied across regions as a result of differential phasing of seasonal influenza across the southeastern and central-northern regions. Specifically, given that seasonal influenza activity typically occurs during winter in central and northern Mexico [40], the pandemic virus started disseminating directly following the circulation of seasonal influenza strains, potentially stimulating cross-subtype immunity and reducing transmission early during the pandemic. In contrast, 6–9 months may have passed since the most recent seasonal influenza activity in the southeastern region where seasonal influenza activity occurred in the summer [41], potentially resulting in populations with relatively lower pre-pandemic immunity levels. In sensitivity analyses, we explored the possibility that pre-pandemic susceptibility varied independently in northern and southern states. A model allowing for regional differences in pre-pandemic immunity performed better than a model without, supporting higher prior immunity in the northern and central states than in the southeastern states; however we have no specific biological information to support this model. Further, it should be noted the model with regional differences in pre-pandemic immunity benefitted from prior information on the spatial structure of the pandemic wave in different Mexican regions, data that were not provided to the other models tested. Overall, the epidemiological consequences of prior immunity to pandemic influenza remains heavily debated and a subject worthy of further experimental and modeling work [36–39,42].

Although our model accurately captured the overall spatial structure of the pandemic, correctly predicting the season of peak pandemic activity in 29 of 32 states, some details of the pandemic were missed. Specifically, in some southeastern states, simulated summer outbreaks lagged 2–4 weeks behind the observed outbreaks. Further, the model did not accurately describe the intense growth of fall outbreaks in states such as Sonora, Tlaxcala and Hidalgo (Fig 4). The lack of age-structure in our model and finer details of the relationship between influenza and environmental forcing could explain these differences. As descriptive analyses revealed high correlation between regional pandemic patterns and specific humidity, we selected specific humidity as the most likely driver of transmission in our mechanistic approach. In line with previous work [20], we did not further consider the putative effect of other environmental covariates, particularly temperature, due to high collinearity with specific humidity. Another possible source of error is that we used weather data corresponding to the largest population in each state. Specific humidity and other weather variables can vary significantly within states. By using weather data at the largest population center in each state we mitigated some of the effects of spatial climate variability, but it should be noted that these data may not accurately represent conditions for people living outside the population center. Further, we did not consider stochastic effects and assumed that these effects were limited due to our focus on the large populations of Mexican states in a pandemic period where incidence is high, following earlier work [43]. An alternative approach would be to do seed infected hosts at critical times prior to each wave as in [44], which would be important to consider for small populations. Another issue in developing meta-population models for Mexico is the lack of detailed mobility data. Although the addition of a hub centered around the greater Mexico city area did not seem to affect the dynamics of the 2009 pandemic, details of population mobility could be more important in inter-pandemic seasons [30]. Future work could concentrate on calibrating more detailed meta-population model to incidence and mobility data in Mexico.

Our results do not support a substantial increase in transmission at low levels of specific humidity, which has been observed in previous studies for both seasonal [1,5,6] and pandemic influenza [20–23]. However, pandemic activity was concentrated during April-October in Mexico when specific humidity levels were at moderate-to-high levels in a majority of states, making it difficult to assess the relationship between low levels of specific humidity and transmission. Another possibility is that—as discussed above—prior immunity in the spring may have been high in the northern (drier states) due to recent seasonal influenza transmission thereby inhibiting the spread of influenza during the period at the beginning of the pandemic when specific humidity was relatively low.

Finally, although we accounted for institutional intervention measures, we did not account for changes in personal behavior (e.g., hand washing, avoiding public spaces, masks) that may have varied across time and space. Indeed, behavior change may have contributed to the formation of multiple waves in the UK during the 1918–1919 influenza pandemic [45]. Limited evidence indicates there were changes in travel behavior in Mexico in response to the pandemic [46]. Changes in social behavior, however, would not explain regional differences in pandemic patterns across Mexico unless these changes were regionally heterogeneous.

Overall, our results indicate that the occurrence of spatially-heterogeneous waves of the A/H1N1 pandemic virus in Mexico can be understood through consideration of local specific humidity conditions, susceptibility, and school-driven mixing patterns. The effect of humidity on pandemic influenza transmission in Mexico is consistent with a recent global model of seasonal influenza activity that stipulates a bimodal relationship between influenza and specific humidity, where transmission is favored by very high and very low levels [1]. Broadly, these findings suggest that a greater understanding of the mechanisms that drive inter-pandemic influenza epidemics may increase our capacity to predict the timing of major outbreaks associated with novel pandemic influenza viruses.

## Supporting Information

S1 Text Additional information regarding methods and results. (DOCX)
