# Supplementary material for: Impact of School Cycles and Environmental Forcing on the Timing of Pandemic Influenza Activity in Mexican States, May-December 2009
Source: PLoS Comput Biol. 2015 Aug 20;11(8):e1004337. doi: 10.1371/journal.pcbi.1004337 (PMC4546376; doi:10.1371/journal.pcbi.1004337)
Supplement: S1 Text — (DOCX) [file pcbi.1004337.s001.docx]

**Supplementary Text**

Impact of school cycles and environmental forcing on the timing of pandemic influenza activity in Mexican states, May-December 2009

1. Supporting Results: Relationship between transmission and specific humidity


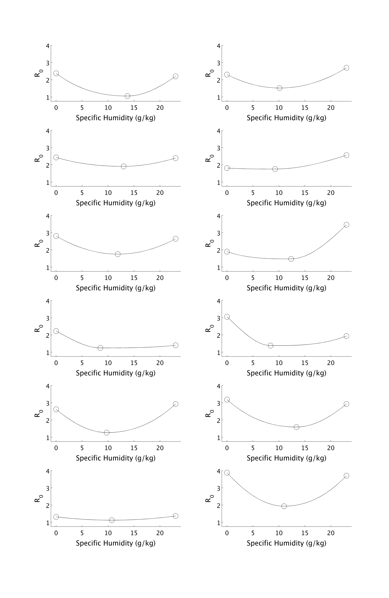
As explained in the main manuscript, we developed a method that allowed *R0* to vary as a function of specific humidity. The method developed allowed this relationship to take many different shapes by defining three critical points and fitting a spline through each point. Below are 10 random relationships using this method.

**Figure S1**. Examples of curves generated by interpolating across points *p1, p2, p3* using the PCHIP method. Note that the curves pass directly through each point without overshooting. This allowed us to explicitly define the bounds of the curves. Further, the method allows for L-, J-, and U-shaped curves, as well as flat lines.

2. Supporting Results: Additional Model Variables

As a sensitivity analysis we allowed several variables that were fixed in the primary models (see main manuscript) to vary: mean latent period, *θ*, mean infectious period, *α,* and initial susceptibility, *μ(0)* (Table S1). For continuity with the main manuscript, below, we focus on the results of model S1 (Table S1), which did not include travel effects. Overall, the models performed well with respect to the AIC and predicting the season of the wave for each state (Table S2). The models reinforced the main conclusions of the manuscript, namely, a small increase in transmission at high levels of specific humidity allowed for a summer wave to affect the humid southeastern states (Table S3); summer break curtailed a summer wave in the drier central and northern states; and a fall wave in the central and northern states was sparked by schools beginning in the fall (Figure S2).

However, the added variables made the model more unstable and did not allow the parameter that controls the increase in transmission at high levels of humidity to converge (Table S3). Indeed, our MCMC estimation algorithm achieved high convergence for model S1 with the exception of parameter *w_4_* which determines the increase in transmission at high levels of specific humidity. However, this parameter was estimated to be well above zero for all models (Table S3) providing strong evidence of increased transmission with increasing specific humidity, consistent with the primary models. Further, the infection attack rates for the best-fit model were higher than expected, averaging 40% across all states and ranging from 26-62%. The models also suggested a low initial susceptibility of approximately 80%, rather than the 95% specified in the primary models which was based on serological surveys. Overall, this sensitivity analysis suggests that fixing the mean latency period, mean infectious period and the initial susceptibility in the primary models did not affect our main conclusions.

| **Parameter** | **Description** | **Refs.** | **Range** | Model S1  (No spatial coupling, independent fit to each state) | Model S2  (meta-population model with coupling with edjacent states) | Model S3  ( meta-population model with coupling with edjacent states and greater Mexico City hub ) |
| --- | --- | --- | --- | --- | --- | --- |
| *w_1_* | Value of *specific humidity* (g/kg) corresponding to minimum *R_0_* | [1] | 6-14 | x | x | x |
| *w_2_* | Minimum *R_0_* | [6] | 1-2 | x | x | x |
| *w_3_* | Added to *w_2_* to define *R_0_* for  *specific humidity* = 0 g/kg | [6] | 0-2 | x | x | x |
| *w_4_* | Added to *w_2_* to define *R_0_* for  *specific humidity* = 23 g/kg | [1] | 0-2 | x | x | x |
| γ*_1_* | Transmission efficiency during school vacation | [15] | 0.55-0.90 | x | x | x |
| γ*_2_* | Transmission efficiency during intervention and school closures | [15, 16] | 0.55-0.90 | x | x | x |
| *τ(0)* | Rate of infected population at time = 0 | NA | 10^-6^ -10^-3^ | x | x | x |
| *c_adj_* | Links force of infection between adjacent states | NA | 0-1 | -- | x | x |
| *c_hub_* | Links force of infection in hub states with all other states | NA | 0-1 | -- | -- | x |
| *μ(0)* | Pre-pandemic population susceptibility fraction | [32] | 0.75 - 0.95 | x | x | x |
| *Θ* | Latency period (days) | [28] | 1.0 - 1.8 | x | x | x |
| α | Mean infectious period (days) | [28, 29] | 0.6 - 2.6 | x | x | x |

**Table S1.** Epidemiological parameters included in each supplementary model. The “x” indicates parameters that are included in the corresponding model.

|  | Summer Wave  Predicted/Observed | Fall Wave  Predicted/Observed | Fall Wave  Predicted/Observed | AIC |
| --- | --- | --- | --- | --- |
| Model S1  (No Travel) | 5/6 (83%) | 24/26 (92%) | 29/32 (91%) | -94231 |
| Model S2  (Adjacent Only) | 4/6 (67%) | 25/26 (96%) | 29/32 (91%) | -93690 |
| Model S3  (Hub and Adjacent) | 5/6 (83%) | 24/26 (92%) | 29/32 (91%) | -94013 |

**Table S2.** Summary of supplementary models and measures of goodness-of-fit.

|  | Model S1  (No Travel) | | Model S2  (Adjacent Only) | | Model S3  (Hub and Adjacent) | |
| --- | --- | --- | --- | --- | --- | --- |
| Parameters | Parameter Estimate  (std) | Geweke | Parameter Estimate  (std) | Geweke | Parameter Estimate  (std) | Geweke |
| *w_1_* | 9.56 (0.12) | 0.68 | 9.26 (0.42) | 0.28 | 8.06 (0.06) | 0.02 |
| *w_2_* | 1.70 (0.07) | 0.99 | 1.28 (0.08) | 0.04 | 1.47 (0.03) | 0.77 |
| *w_3_* | 0.11 (0.07) | 0.86 | 0.32 (0.13) | 0.58 | 0.11 (0.01) | 0.90 |
| *w_4_* | 0.22 (0.06) | 0.33 | 0.67 (0.08) | 0.50 | 0.15 (0.01) | 0.34 |
| γ*_1_* | 0.78 (0.03) | 0.97 | 0.81 (0.03) | 0.91 | 0.85 (0.01) | 0.97 |
| γ*_2_* | 0.62 (0.07) | 0.88 | 0.81 (0.08) | 0.81 | 0.83 (0.01) | 0.92 |
| *τ(0)* | 6.18e-5 (1.27e-6) | 0.98 | 2.2e-5  (1.5e-6) | 0.78 | 2.4e-5  (5.6e-7) | 0.93 |
| *c_adj_* | -- | -- | 0.05 (0.01) | 0.61 | 0.03 (0.01) | 0.66 |
| *c_hub_* | -- | -- | -- | -- | 7.3e-4 (3.8e-4) | 0.11 |
| *μ(0)* | 0.78 (0.03) | 0.97 | 0.86 (0.08) | 0.99 | 0.82 (0.02) | 0.90 |
| *Θ* | 2.27 (0.04) | 0.94 | 0.62 (0.14) | 0.35 | 0.73 (0.02) | 0.97 |
| α | 1.64 (0.04) | 0.94 | 0.62 (0.05) | 0.86 | 0.62 (0.03) | 0.85 |

**Table S3.** Epidemiological parameter estimates and ranges for supplementary models.


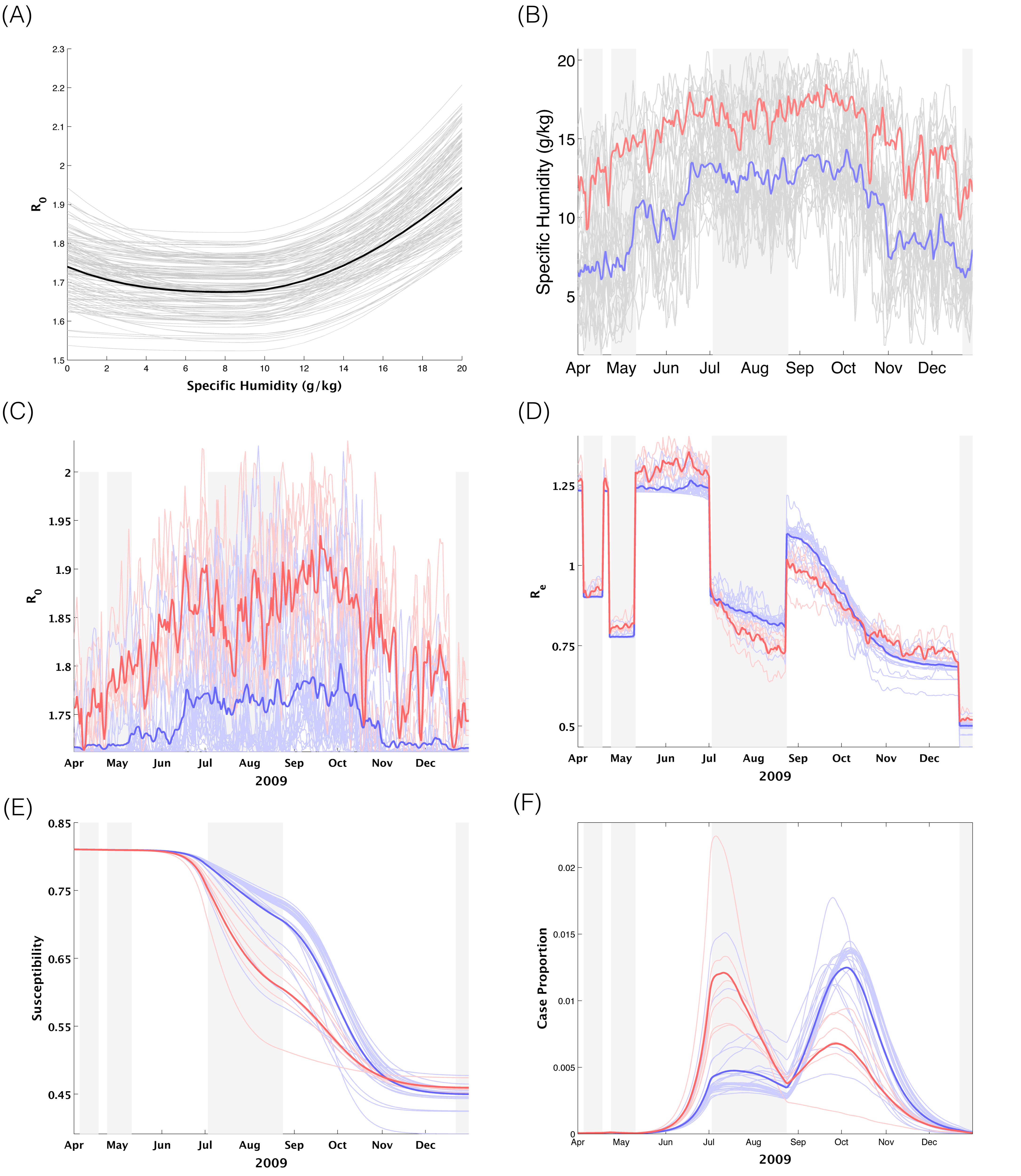


**Figure S2**. Variability of *R_0_* and *R_e_* during May-December 2009 as a function of changes in specific humidity, interventions, school cycles, and susceptibility, and the resulting impact on pandemic influenza activity, as predicted by the model. (*A*) The relationship between specific humidity and *R_0_*. The gray lines are 500 samples generated from the posterior distributions, and the black line corresponds to the mean value of the posterior means. (*B*) The time series of average specific humidity for central and northern states (solid line) and southeastern states (dashed line). (*C*) Time series of *R_0_* for best-fit parameter combination. The step features are related to spring break, the intervention period, summer break and winter break, respectively. (*D*) Time series of simulated *R_e_*. (*E*) Time series of simulated population level susceptibility. (*F*) Time series of simulated case proportions. For *B-F*, shaded areas in background correspond to spring vacation, period of school closures and intervention measures, summer vacation, and winter vacation, respectively. The solid lines correspond to the central and northern states, and the dashed lines correspond to the southeastern states. The simulated values were generated using the mean value of the posterior means for each parameter.

3. Supporting Results: Modified model (removed summer vacation)

To illustrate the effect of summer vacation (for school students) on the progression of the pandemic across Mexico, we simulated the pandemic without a summer vacation period. Specifically, we evaluated the specific humidity based model using the posterior means of each parameter while imposing that γ*_1_* and γ*_2_* to be equal during the summer vacation period.

The modified model suggests that without a summer vacation period reducing transmission rates, the early summer outbreaks in the southeastern states would have been followed by outbreaks in the central and northern states within 3.5 weeks (Figure S3). This suggests that, in of itself, specific humidity had a relatively minor effect on the different timing of the outbreaks. However, the summer vacation amplified the small difference in transmission (and the small difference in timing of the outbreaks) by reducing transmission in the summer and delaying outbreaks in central and northern states until students returned in the fall.

**Figure S3**. Simulated case proportions for central and northern states (solid line) and southeastern states (dashed line) for a scenario in which there was no summer vacation. The model suggests that the peaks in pandemic influenza activity for the two regions would have only been 2-3 weeks apart.

4. Supporting Methods and Results: Modified model (initial susceptibility varies across regions)

We assessed the possibility that population-level susceptibility varied across northern and central states, and southeastern states. Specifically we allowed susceptibility in the central and northern states that experienced the fall wave, *μ_n_,* and susceptibility in the southeastern states, *μ_s_,* to vary independently between 0.75 and 0.95. Altogether, the effective reproduction number became:

It should be noted that this model is provided an advantage over other models given that the states were grouped based on the timing (i.e., summer or fall) of the observed pandemic wave in each state. For all other models, the model parameters were constant across states and regions. Indeed, this modified model significantly outperformed the specific-humidity based models described in the manuscript. The model predicted the peak timing of the pandemic waves (summer or fall) in all states, and its AIC was -94834. Estimates for susceptibility in northern and southern states was 0.81 and 0.86, respectively (see Table S3). Although there is no empirical evidence that susceptibility varied across the region, these results suggest that this is a viable explanation of the different patterns and should be investigated further.

| **Parameter** | **Description** | **Range/values** | **Best-fit parameter estimates (95% CI)** | **Geweke** |
| --- | --- | --- | --- | --- |
| *R_0_* | Basic reproduction number | 1-2 | 1.51 (1.35, 1.70) | 0.96 |
| γ*_1_* | Transmission efficiency during school vacation | 0.55-0.90 | 0.83 (0.78,0.89) | 0.99 |
| γ*_2_* | Transmission efficiency during intervention and school closures | 0.55-0.90 | 0.78 (0.65, 0.88) | 0.92 |
| *τ(0)* | Rate of infection at time 0 | 10^-6^ -10^-3^ | 8.78 x 10^-6^  (5.50 x 10^-6^, 1.09 x 10^-5^) | 0.37 |
| *μ_n_(0)* | Pre-pandemic population susceptibility fraction for northern states | 0.55-0.95 | 0.85 (0.78, 0.93) | 0.96 |
| *μ_s_(0)* | Pre-pandemic population susceptibility fraction for southern states | 0.55-0.95 | 0.90 (0.82, 0.99) | 0.96 |
| *Θ* | Latency period (days) | 1.4 | Fixed | NA |
| α | Mean infectious period (days) | 1.6 | Fixed | NA |

**Table S3**. Epidemiological parameter estimates for model allowing for differences in susceptibility between southern and northern states.
